# The association between toxic leadership behaviors and nurses’ career resilience: heterogeneous indirect roles of regulatory focus profiles

**DOI:** 10.3389/fpsyg.2026.1951423

**Published:** 2026-09-07

**Authors:** Yue Gu, Hang Liu, Tingyao Nie, Ying Li, Xiaozhen Sun, Rui Yang, Yanping Qi, Liwen Guo, Liang Tong

**Affiliations:** 1 Department of Urology, First Affiliated Hospital of Zhengzhou University, Zhengzhou, China; 2 Department of Geriatrics, The First Affiliated Hospital of Hunan University of Chinese Medicine, Changsha, China; 3 Department of Nursing, First Affiliated Hospital of Zhengzhou University, Zhengzhou, China; 4 Department of Nursing, Hunan Cancer Hospital, Changsha, China; 5 Department of Medical Oncology, Shenzhen Hospital (Futian) of Guangzhou University of Chinese Medicine, Shenzhen, China

**Keywords:** career resilience, latent profile analysis, nurses, regulatory focus, toxic leadership behaviors

## Abstract

**Background:**

Toxic leadership behaviors by nurse managers may be associated with lower career resilience among nurses. However, it remains unclear whether different regulatory focus profiles exhibit heterogeneous indirect associations in this context.

**Aim:**

To examine the association between toxic leadership behaviors and nurses’ career resilience, and to investigate whether different regulatory focus profiles exhibit distinct indirect associations in this relationship.

**Methods:**

From May to October 2025, a multicenter cross-sectional survey was conducted among 1,210 nurses using the Toxic Leadership Behaviors of Nurse Managers Scale, the Nurse Regulatory Focus Scale, and the Career Resilience Scale. Latent profile analysis identified regulatory focus profiles, and three-step methods were used to examine profile differences and associations while accounting for classification uncertainty. Profile-based path analysis estimated overall and profile-specific statistical indirect associations.

**Results:**

Four regulatory focus profiles were identified: Low-Regulatory (9.8%), Balanced (40.4%), Prevention-Dominant (11.9%), and Dual-High (37.9%). Career resilience was highest in the Dual-High profile and lowest in the Low-Regulatory profile. Higher toxic leadership scores were associated with lower odds of membership in the other three profiles relative to the Low-Regulatory profile. Toxic leadership was negatively associated with career resilience (total: *β* = −0.388, 95% CI [−0.439, −0.344]; direct: *β* = −0.280, 95% CI [−0.325, −0.241]), with a negative overall indirect association through regulatory focus profiles (*β* = −0.108, 95% CI [−0.145, −0.082]). Relative to the Low-Regulatory profile, the relative indirect contribution was negative for the Dual-High profile (*β* = −0.163, 95% CI [−0.228, −0.116]), positive for the Prevention-Dominant profile (*β* = 0.065, 95% CI [0.040, 0.100]), and nonsignificant for the Balanced profile.

**Conclusion:**

Toxic leadership behaviors were negatively associated with nurses’ career resilience, and the profile-specific relative indirect contributions varied across regulatory focus profiles.

## 1 Background

Psychological health and professional sustainability have become key issues for nurses worldwide (World Health Organization, 2025; Zabin et al., 2023; Pi et al., 2025). As frontline professionals in the healthcare sector, nurses are often confronted with excessive workloads, complex clinical situations, and occupational stress—factors that have long put their career resilience to the test (Zabin et al., 2023; Pi et al., 2025). General resilience is understood as the comprehensive ability to cope with adversity and adapt to various life situations (Fletcher and Sarkar, 2013). Career resilience, on the other hand, is more specific and refers exclusively to the professional context. Within the framework of career motivation theory, career resilience reflects a person’s general ability to adapt to an ever-changing work environment, to overcome professional challenges, and to continue to grow professionally even under demanding working conditions (London, 1983; London and Noe, 1997). Since career resilience develops and manifests itself within a specific professional context, it may be associated with both personal traits and situational factors such as involvement in decision-making processes, support from management, and opportunities for professional development (London, 1983; London and Noe, 1997; London, 1993). Previous studies involving nurses show that high career resilience is associated with a lower turnover rate and positive occupational outcomes, such as high-quality care (Montgomery and Patrician, 2022; Vaisi-Raygani et al., 2025). Furthermore, career resilience enables nurses to adapt flexibly to a high-stress clinical environment and to perform their duties effectively even under work-related pressure (Gagnon-Béland et al., 2024; Shalom et al., 2025). Since career resilience is directly linked to individual adaptability as well as persistence in professional development and growth (London, 1983; London and Noe, 1997), identifying the organizational and psychological factors associated with career resilience can provide more concrete insights into how to retain nurses in the organization over the long term and maintain their occupational functioning.

Among organizational factors, leadership behaviors are important organizational factors associated with nurses’ well-being and job satisfaction (Niinihuhta and Häggman-Laitila, 2022). In contrast to supportive and transformational leadership styles, toxic leadership behaviors are destructive management behaviors characterized by grandiosity, narcissism, self-aggrandizement, and the denigration of others (Labrague et al., 2020). Previous research has shown that toxic leadership behaviors are associated with numerous negative workplace outcomes, particularly lower job satisfaction and increased turnover intention among nursing staff (Ofei et al., 2023; Labrague, 2024). Toxic and abusive leadership behaviors are associated with poor mental health, increased work stress, and a stronger desire to leave healthcare organizations and the nursing profession (Labrague, 2024; Lavoie-Tremblay et al., 2016). According to the Conservation of Resources Theory, prolonged exposure to stressful work environments depletes an individual’s valuable psychological resources, triggering a process of resource depletion that, in turn, reduces their ability to cope with subsequent sources of work-related stress (Hobfoll, 2001). Although previous research has focused primarily on outcome measures such as job satisfaction, mental health, intent to leave, work performance, and patient safety (Ofei et al., 2023; Labrague, 2024), there is still insufficient evidence regarding the psychological link between toxic leadership behaviors and the career resilience of nurses (Ofei et al., 2023; Labrague, 2024).

Furthermore, this potential psychological pathway may vary across nurses. Even nurses facing similar working conditions may react differently depending on their motivational orientation and how they cope with the demands of the work environment (Higgins, 1997; Wallace et al., 2009). Regulatory Focus Theory offers a useful theoretical framework for understanding these individual differences (Higgins, 1997). According to this theory, people regulate their behavior through two motivational systems: promotion focus, which emphasizes growth, performance, and progress, and prevention focus, which prioritizes safety, responsibility, and the avoidance of mistakes and losses (Higgins, 1997). In an organizational context, leaders’ behaviors can serve as prominent environmental signals that help shape subordinates’ motivational orientations, including their regulatory focus (Hamstra et al., 2014; Johnson et al., 2021). Therefore, toxic leadership behaviors, such as humiliation, narcissism, excessive ego, and inappropriate behavior (Labrague et al., 2020), may serve as negative signals in the workplace and be associated with how nurses perceive their professional challenges and their ability to manage them (Labrague et al., 2020; Labrague, 2024; Johnson et al., 2021). In contrast, individuals with a strong promotion focus tend to concentrate on professional development, performance, and expected outcomes, while those with a strong prevention focus direct their attention toward safety, responsibility, and the avoidance of negative outcomes (Higgins, 1997; Wallace et al., 2009). Among nurses, regulatory focus has been associated with burnout, with perceptions of transformational leadership mediating this relationship (Shi et al., 2015); furthermore, regulatory focus has been associated with team job crafting (Wang et al., 2026). Overall, these findings suggest that regulatory focus may represent a psychological pathway through which toxic leadership behaviors are associated with nurses’ career resilience (Higgins, 1997; Johnson et al., 2021; Shi et al., 2015; Wang et al., 2026).

It remains unclear whether nurses’ regulatory focuss differ in various ways, or what role these characteristics play in the relationship between toxic leadership behaviors and career resilience. Traditional variable-centered mediation analyses typically estimate indirect relationships at the population level, which can lead to ambiguities regarding the diversity observed across different subgroups (Muthén and Muthén, 2000; Wang et al., 2020). In contrast, latent profile analysis (LPA) is a person-centered approach that identifies subgroups based on how promotion and prevention focus co-occur within individuals (Spurk et al., 2020). Unlike variable-centered analyses, which estimate the average and independent associations of the two regulatory-focus dimensions, LPA can characterize empirically occurring joint patterns that differ in their overall level, relative configuration, or both. By combining LPA with profile-based indirect association analysis, researchers can examine whether these joint patterns are associated with different indirect associations between toxic leadership behaviors and career resilience (Wang et al., 2020). This approach allows for a more detailed examination of whether such subgroups exist (Wang et al., 2020). It contributes to a deeper understanding of the psychological mechanisms underlying the relationship between toxic leadership behaviors and career resilience among nurses, taking into account finer nuances. The main objective of this study is therefore to examine various indirect relationships between toxic leadership behaviors and nurses’ career resilience based on the characteristics of regulatory focus. To achieve this objective, we first identified the characteristics of nurses’ regulatory focus and then quantified the indirect relationships between toxic leadership behaviors and career resilience. Understanding these diverse psychological patterns provides a theoretical foundation for developing targeted nursing management strategies that will support nurses’ long-term career resilience.

## 2 Methods

### 2.1 Study design

This study was conducted as a multicenter cross-sectional study. The research report was prepared in accordance with the Strengthening the Reporting of Observational Studies in Epidemiology (STROBE) guidelines for cross-sectional studies (von Elm et al., 2007). By combining LPA with profile-based path analysis, the study examined statistical indirect associations between toxic leadership behaviors and career resilience through regulatory focus profile membership.

### 2.2 Participants and recruitment

In this study, convenience sampling was used. Participants were recruited through a multicenter online survey conducted between May and October 2025. They came from six hospitals located in the Chinese provinces of Henan, Hunan, and Guangdong. Participating institutions included four tertiary Grade A general hospitals and two tertiary Grade A specialist hospitals. Participating clinical departments included internal medicine, surgery, intensive care, and other clinical departments. After obtaining consent from the participating hospitals, the research coordinator sent the questionnaire link electronically. All participants provided informed consent after receiving a clear explanation prior to completing the questionnaire. Participants had to be registered nurses with a valid nursing license and at least 1 year of clinical experience; nursing interns and nurses not practicing in a clinical setting were excluded from this study. The one-year clinical experience criterion was established to enable participants to provide informed assessments of recurring leadership behaviors and professional responses, ensuring that they had sufficient experience with nursing managers in their current clinical employment settings. Since nursing interns had not yet assumed the full professional responsibilities of registered nurses, they were excluded. Meanwhile, non-clinical nurses were also excluded, as they did not regularly interact with nursing managers in direct care settings.

To maintain data quality, a questionnaire was considered invalid if it met any of the following criteria: (1) the completion time was less than 120 s; (2) at least 90% of the scale items (43 items or more) had identical responses; (3) duplicate submissions were identified using device information or a combination of IP address, identical sociodemographic information, and submission time within 24 h; or (4) responses did not meet the eligibility criteria. Two researchers independently reviewed the collected data and resolved all discrepancies through discussion. Previous simulation studies have shown that sample size, the number of indicators and the degree of separation between latent profiles affect the performance of LPA (Tein et al., 2013). According to the methodological recommendations on the sample size required to detect indirect effects (Fritz and MacKinnon, 2007), the initial target was set at 300 to 500 participants. Ultimately, this study included 1,210 participants, well above the initial target, providing an ample sample for the analysis. All items in the online questionnaire were mandatory; therefore, there were no missing data among the 1,210 valid responses included in the analysis.

### 2.3 Measures

#### 2.3.1 Sociodemographic and occupational characteristics

A sociodemographic and occupational questionnaire designed by the researchers was used to collect sociodemographic and occupational information from the study participants. The questionnaire covered age, gender, highest educational level, parental status, years of nursing experience, number of night shifts per month, current clinical department, and physical exercise. The specific classifications are as follows: age was categorized as <30 years and ≥30 years; gender as male and female; educational level as associate degree, bachelor’s degree, and master’s degree or above; whether or not the participant had children as “yes” and “no”; nursing experience as ≤5 years, 6–9 years, and ≥10 years; monthly night shifts as 0, 1–3, 4–6, and ≥7; clinical department as surgical, medical, critical care unit, and other departments; and physical exercise as never, occasionally, and regularly.

#### 2.3.2 Toxic leadership behaviors of nurse managers scale (ToxBH-NM)

The ToxBH-NM was used to assess nurses’ perceptions of toxic leadership behaviors exhibited by their managers. The scale was originally developed by Labrague et al. (2020) and underwent psychometric validation specifically in a nursing population. It was subsequently culturally adapted and psychometrically evaluated among Chinese nurses by Yao et al. (2023). The scale comprises 30 items across four dimensions: authoritarian behavior (items 1–15), narcissistic behavior (items 16–24), self-promotional behavior (items 25–27), and abusive behavior (items 28–30). Each item is rated on a 5-point Likert scale ranging from 1 (“not at all”) to 5 (“very often”), yielding a total score of 30–150. Higher scores indicate greater perceived toxic leadership behaviors among nurse managers. The original scale demonstrated good internal consistency, with a Cronbach’s α coefficient of 0.975 (Labrague et al., 2020). In the present study, Cronbach’s α was 0.982 for the overall scale and 0.976, 0.926, 0.911, and 0.922 for the authoritarian behavior, narcissistic behavior, self-promotional behavior, and abusive behavior subscales, respectively.

#### 2.3.3 Nurse regulatory focus scale (NRFS)

The NRFS was adapted for Chinese nurses from the Regulatory Focus at Work Scale developed by Wallace et al. (2009) and subsequently revised by Xu et al. (2023). The revision was conducted specifically considering the characteristics of nursing work and organizational settings, and its psychometric properties were evaluated in a sample of 1,456 clinical nurses in China. The scale was used to assess nurses’ regulatory focus characteristics. It comprises 12 items divided into two dimensions—promotion focus and prevention focus—with six items in each dimension. All items are rated on a 5-point Likert scale ranging from 1 (“strongly disagree”) to 5 (“strongly agree”). Higher scores on each dimension indicate a stronger orientation toward the corresponding regulatory focus. The Chinese version demonstrated good internal consistency, with Cronbach’s α coefficients of 0.962, 0.929, and 0.970 for the total scale, promotion focus dimension, and prevention focus dimension, respectively (Xu et al., 2023). In the present study, Cronbach’s α coefficients were 0.882 for the overall scale, 0.775 for promotion focus, and 0.850 for prevention focus.

#### 2.3.4 Career resilience scale (CRS)

The CRS was used to assess nurses’ career resilience. The scale was based on the career resilience construct proposed within London’s career motivation framework (London, 1993) and was subsequently adapted for use among Chinese knowledge workers by Wang (Wang, 2015). The scale comprises five items rated on a 5-point Likert scale ranging from 1 (“strongly disagree”) to 5 (“strongly agree”). The total score ranges from 5 to 25, with higher scores indicating greater career resilience. The Chinese version demonstrated acceptable internal consistency, with a Cronbach’s α coefficient of 0.826 (Wang, 2015); in the present nursing sample, Cronbach’s α was 0.800, indicating acceptable internal consistency.

### 2.4 Ethical considerations

This study was approved by the Institutional Review Board of the First Affiliated Hospital of Zhengzhou University (Approval No. 2025-KY-1990-001). The study was conducted in accordance with the principles of the Declaration of Helsinki.

### 2.5 Data analysis

All statistical analyses were performed using R version 4.3.2. Categorical variables were presented as frequencies and percentages [*n* (%)]. Continuous variables were expressed as mean ± standard deviation (M ± SD). Pearson’s correlation analysis was used to examine the relationship between nurses’ toxic leadership behaviors, regulatory focus, and career resilience. Independent-samples t-tests were used to examine differences in career resilience among nurses with different sociodemographic and occupational characteristics, and for categorical variables with three or more categories, one-way analysis of variance (ANOVA) was used. When the assumption of homogeneity of variances was not met, Welch’s *t*-test or Welch’s ANOVA was used, depending on the variable’s categorization. When a statistically significant overall difference was found among multiple groups, pairwise *post hoc* comparisons were also conducted using the Games–Howell method.

LPA was conducted using the mclust package (Scrucca et al., 2016). To detect subtle heterogeneities that might be masked by composite scale scores, the 12 items of the Nurse Regulatory Focus Scale were used as continuous indicators. Gaussian mixture models were estimated for one to five profiles. An unconstrained covariance model was used for the single-profile solution, while EEV parameterization was adopted for solutions involving two to five profiles. The models were estimated using the expectation-maximisation (EM) algorithm (Scrucca et al., 2016). To assess sensitivity to initialization and reduce the risk of local maxima, a random-start sensitivity analysis was conducted using 500 independent random initializations for each two- to five-profile candidate solution (Spurk et al., 2020). EM convergence was assessed using a relative log-likelihood tolerance of 1 × 10^−5^. Replication of the best log-likelihood solution was evaluated across independent random initializations; solutions with a relative log-likelihood difference of ≤1 × 10^−6^ from the maximum were considered replications of the best solution. Model selection took into account the Akaike information criterion (AIC), Bayesian information criterion (BIC), sample-size-adjusted Bayesian information criterion (aBIC), entropy, Lo–Mendell–Rubin likelihood ratio test (LMRT), bootstrap likelihood ratio test (BLRT), profile size, numerical stability, parsimony, and substantive interpretability (Spurk et al., 2020; Nylund et al., 2007). Profiles accounting for less than 5 per cent of the sample were considered potentially unstable and were interpreted with caution (Spurk et al., 2020).

We compared career resilience across profiles using the Bolck–Croon–Hagenaars (BCH) three-step approach (Bolck et al., 2004). These comparisons included an overall comparison and pairwise comparisons corrected using Holm’s method. The associations with profile membership were examined using error-corrected univariable and multivariable multinomial logistic regression analyses, and the Low-Regulatory profile was adopted as the reference category. Uncertainty in classification was addressed using an error-corrected three-step procedure (Bolck et al., 2004; Vermunt, 2010). Covariates were selected based on previous literature and preliminary analyses examining their associations with career resilience or regulatory focus profile membership.

Using profile-based path analysis, while taking into account uncertainty in classification, we examined the statistical indirect associations between toxic leadership behaviors and career resilience through regulatory focus profile membership (Wang et al., 2020; Bolck et al., 2004; Vermunt, 2010). Profile membership was treated as a nominal categorical variable, with the Low-Regulatory profile used as the reference category. The relationship between profile membership and career resilience was examined using the BCH three-step approach (Bolck et al., 2004), which accounts for classification uncertainty when evaluating distal outcomes. The total association was decomposed into direct and overall statistical indirect associations, and the latter was further decomposed into profile-specific relative indirect contributions (Wang et al., 2020). These relative indirect contributions were estimated using a model-standardization procedure within the BCH three-step framework. Toxic leadership was treated as a standardized continuous predictor, and contributions were calculated by comparing model-estimated profile membership probabilities across a −0.5 SD to +0.5 SD toxic leadership contrast and combining these probability changes with BCH-adjusted career resilience contrasts between each profile and the Low-Regulatory reference profile. Bootstrap standard errors and percentile 95% confidence intervals (CIs) were calculated based on 5,000 bootstrap resamples, in which BCH weights were recalculated and the error-corrected multinomial and BCH outcome models were re-estimated conditional on the original latent profile solution (Preacher and Hayes, 2008). These procedures were implemented in R 4.3.2 using custom R functions.

## 3 Results

### 3.1 Sociodemographic and occupational characteristics

A total of 1,210 nurses participated in the study. Of these, 726 (60.00%) were younger than 30 years, 1,043 (86.20%) were female, and 1,010 (83.47%) held a bachelor’s degree. In addition, 880 participants (72.73%) had children, and 618 (51.07%) had 5 years or less of nursing experience. Regarding work characteristics, 480 nurses (39.67%) reported working seven or more night shifts per month, and 439 (36.28%) worked in surgical departments. Regarding physical exercise, 609 participants (50.33%) reported exercising occasionally. Detailed sociodemographic and occupational characteristics are presented in Table 1.

**Table 1:** Sociodemographic and occupational characteristics and comparisons of career resilience among participants (*n* = 1,210).

Characteristics	*n* (*%*)	Career resilience, M ± SD	Test statistic	*p*	Effect size
Age (years)			−5.218^a^	<0.001	−0.306^d^
<30	726 (60.00%)	20.16 ± 3.50			
≥30	484 (40.00%)	21.20 ± 3.20			
Gender			−0.759^a^	0.448	−0.063^d^
Male	167 (13.80%)	20.39 ± 3.96			
Female	1,043 (86.20%)	20.61 ± 3.33			
Education level			4.720^b^	0.009	0.008^e^
Associate degree	88 (7.27%)	21.00 ± 3.55			
Bachelor’s degree	1,010 (83.47%)	20.64 ± 3.31			
Master’s degree or above	112 (9.26%)	19.68 ± 4.12			
Parental status			−0.036^a^	0.971	−0.002^d^
Yes	880 (72.73%)	20.57 ± 3.48			
No	330 (27.27%)	20.58 ± 3.29			
Nursing experience (years)			29.298^c^	<0.001	0.042^e^
≤5	618 (51.07%)	19.98 ± 3.58			
6–9	228 (18.84%)	20.56 ± 3.32			
≥10	364 (30.08%)	21.59 ± 2.95			
Monthly night shifts			2.557^b^	0.054	0.006^e^
0	220 (18.18%)	20.93 ± 3.75			
1–3	181 (14.96%)	20.76 ± 3.13			
4–6	329 (27.19%)	20.17 ± 3.73			
7 or more	480 (39.67%)	20.63 ± 3.12			
Clinical department			0.378^b^	0.769	0.001^e^
Surgical	439 (36.28%)	20.52 ± 3.48			
Medical	352 (29.09%)	20.50 ± 3.40			
Critical care units	233 (19.26%)	20.61 ± 3.62			
Other departments	186 (15.37%)	20.81 ± 3.08			
Physical exercise			8.056^c^	<0.001	0.013^e^
Never exercises	467 (38.60%)	20.11 ± 3.68			
Exercises occasionally	609 (50.33%)	20.80 ± 3.27			
Exercises regularly	134 (11.07%)	21.21 ± 2.95			

a Independent-samples *t*-test.

b One-way ANOVA.

c Welch’s ANOVA.

d Cohen’s d.

e Eta squared (*η*^2^).

### 3.2 Associations between career resilience and other variables

The career resilience, regulatory focus, and toxic leadership behavior means and standard deviations in this study were 20.58 ± 3.42, 50.43 ± 7.25, and 66.29 ± 28.15, respectively. Toxic leadership behavior scores had a median of 62.00 (IQR, 39.00–90.00) and ranged from 30 to 142. As shown in Table 2, career resilience was strongly and positively related to regulatory focus (*r* = 0.703, *p* < 0.001). Toxic leadership behaviors were moderately and negatively correlated with career resilience (*r* = −0.423, *p* < 0.001) and regulatory focus (*r* = −0.494, *p* < 0.001). Significant differences in career resilience were observed according to age, educational level, nursing experience, and physical exercise (Table 1).

**Table 2:** Descriptive statistics and correlations among career resilience, regulatory focus, and toxic leadership behaviors.

Variables	M ± SD	Career resilience	Regulatory focus	Toxic leadership behaviors
Career resilience	20.58 ± 3.42	1		
Regulatory focus	50.43 ± 7.25	0.703^***^	1	
Toxic leadership behaviors	66.29 ± 28.15	−0.423^***^	−0.494^***^	1

M = mean; SD = standard deviation.^***^p < 0.001.

### 3.3 Latent profile analyses of regulatory focus

To identify subtle heterogeneity in nurses’ regulatory focus configurations that might be hidden by aggregated dimension scores, each of the 12 items of the Nurse Regulatory Focus Scale was treated as a continuous indicator and included in the latent profile analysis. As shown in Table 3, as the number of profiles increased, the AIC, BIC, and aBIC values decreased, while the LMRT and BLRT remained statistically significant. Although the information-criterion values for the five-profile solution were lower, the four-profile solution had the highest entropy (0.962), indicating greater classification precision. The four profiles accounted for 9.8% to 40.4% of the total sample. Furthermore, the fifth profile did not show a clearly distinct regulatory focus configuration and mainly represented a subdivision of an existing profile, providing limited substantive gain. For the four-profile solution, the highest log-likelihood was successfully replicated across different random starts, supporting the stability of the retained model. Considering classification precision, profile size, substantive interpretability, parsimony, and numerical stability, we retained the four-profile solution (Table 3).

**Table 3:** Fit statistics for latent profile models of regulatory focus.

Model	No. of parameters	AIC	BIC	aBIC	Entropy	LMRT	BLRT	Class proportions
C1	90	31251.545	31710.399	31424.523	—	—	—	1
C2	169	29725.870	30587.496	30050.684	0.922	<0.001	<0.001	0.572/0.428
C3	248	28245.138	29509.535	28721.787	0.902	<0.001	<0.001	0.502/0.092/0.406
C4	327	27582.511	29249.680	28210.996	0.962	<0.001	<0.001	0.098/0.404/0.119/0.379
C5	406	25936.869	28006.810	26717.191	0.940	<0.001	<0.001	0.267/0.299/0.104/0.262/0.068

**Figure 1:**
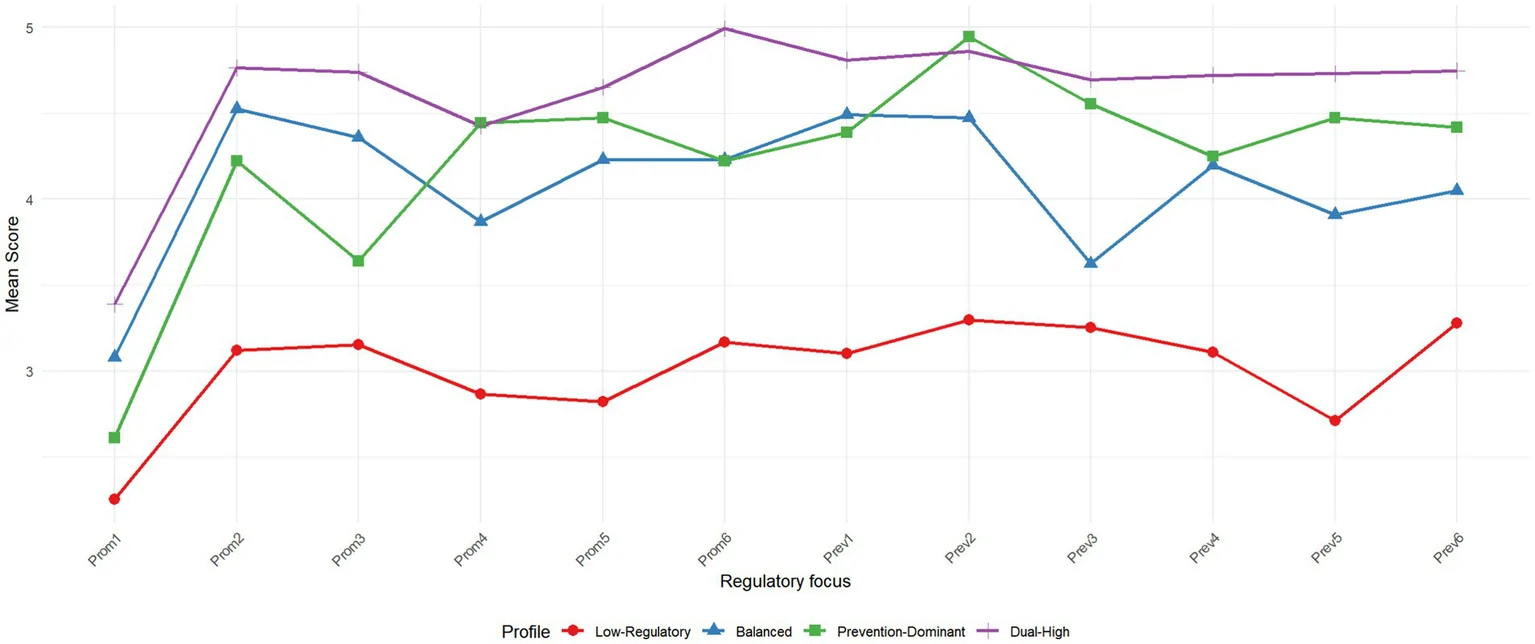
Latent profiles of regulatory focus among nurses.

In Profile 1, participants had relatively low scores on both the promotion focus items (items 1–6) and the prevention focus items (items 7–12), which is why this profile was named the “Low-Regulatory” profile. Profile 2 was named the “Balanced” profile because the levels of promotion focus and prevention focus were relatively balanced. In the third profile, the prevention focus score was significantly higher than the promotion focus score, which is why it was named the “Prevention-Dominant” profile. The fourth profile was called the “Dual-High” profile because it had the highest scores on both promotion focus and prevention focus (Figure 1 and Table 4).

**Table 4:** Descriptive characteristics of regulatory focus dimensions across the identified latent profiles.

Variables	Low-regulatory	Balanced	Prevention-dominant	Dual-high
	*n* = 118	*n* = 489	*n* = 144	*n* = 459
Promotion focus	17.38 ± 4.26	24.29 ± 2.85	23.61 ± 2.79	26.96 ± 2.83
Prevention focus	18.75 ± 4.03	24.74 ± 2.43	27.03 ± 1.85	28.56 ± 2.30

Values are presented as mean ± standard deviation.

### 3.4 Differences in career resilience across regulatory focus profiles

Using a BCH three-step approach to account for uncertainty in latent profile classification, we compared career resilience across four regulatory focus profiles. There was a significant difference in career resilience among the four profiles (Wald *χ*^2^(3) = 398.72, *p* < 0.001). The mean career resilience score calculated using the BCH method was highest for the Dual-High profile (*M* = 22.64, 95% CI [22.33, 22.94]), followed by the Prevention-Dominant profile (*M* = 21.25, 95% CI [20.90, 21.59]) and the Balanced profile (*M* = 19.80, 95% CI [19.54, 20.05]); the Low-Regulatory profile had the lowest mean score (*M* = 15.77, 95% CI [15.08, 16.45]). After Holm adjustment for pairwise comparisons, all six profile differences were statistically significant (*p* < 0.001).

### 3.5 Associations of toxic leadership behaviors with regulatory focus profile membership

Error-corrected univariable multinomial logistic regression analyses showed that age, educational level, nursing experience, monthly night-shift frequency, and toxic leadership behaviors were significantly associated with one or more regulatory focus profile contrasts (Supplementary Table S1). These variables were then entered simultaneously into an error-corrected multivariable multinomial logistic regression model, with the Low-Regulatory profile as the reference category. Higher scores for toxic leadership behaviors were significantly associated with lower odds of membership in the Balanced profile (OR = 0.945, 95% CI [0.934, 0.956]), the Prevention-Dominant profile (OR = 0.958, 95% CI [0.946, 0.971]), and the Dual-High profile (OR = 0.935, 95% CI [0.923, 0.946]); all *p* < 0.001 (Table 5).

**Table 5:** Multivariable multinomial logistic regression analysis of factors associated with regulatory focus profile membership.

Variable	Balanced vs low-regulatory			Prevention-dominant vs low-regulatory		
	*B*	Wald	OR (95% CI)	*B*	Wald	OR (95% CI)
Age (years)						
<30	Ref.			Ref.		
≥30	0.366	0.43	1.442 (0.482–4.309)	1.333^*^	5.03	3.792 (1.183–12.150)
Education level						
Associate degree	Ref.			Ref.		
Bachelor’s degree	−0.494	1.21	0.610 (0.253–1.471)	−1.720^***^	11.32	0.179 (0.066–0.488)
Master’s degree or above	−1.435^**^	6.93	0.238 (0.082–0.693)	−1.806^**^	8.62	0.164 (0.049–0.549)
Nursing experience (years)						
≤5	Ref.			Ref.		
6–9	0.074	0.04	1.077 (0.496–2.338)	0.382	0.75	1.465 (0.616–3.486)
≥10	0.516	0.61	1.676 (0.458–6.125)	0.126	0.03	1.135 (0.283–4.548)
Monthly night shifts						
0	Ref.			Ref.		
1–3	−0.373	0.88	0.689 (0.316–1.503)	−1.181^*^	4.06	0.307 (0.097–0.968)
4–6	0.315	0.73	1.370 (0.666–2.819)	0.083	0.03	1.087 (0.446–2.647)
7 or more	0.292	0.56	1.338 (0.624–2.871)	1.504^***^	11.31	4.501 (1.873–10.815)
Toxic leadership behaviors	−0.057^***^	92.47	0.945 (0.934–0.956)	−0.043^***^	43.73	0.958 (0.946–0.971)

Variable	Dual-high vs low-regulatory		
	*B*	Wald	OR (95% CI)
Age (years)			
<30	Ref.		
≥30	1.236^*^	4.55	3.443 (1.105–10.723)
Education level			
Associate degree	Ref.		
Bachelor’s degree	−1.940^***^	19.13	0.144 (0.060–0.343)
Master’s degree or above	−3.984^***^	40.4	0.019 (0.005–0.064)
Nursing experience (years)			
≤5	Ref.		
6–9	−0.102	0.06	0.903 (0.394–2.072)
≥10	0.441	0.42	1.554 (0.408–5.913)
Monthly night shifts			
0	Ref.		
1–3	−0.729	2.93	0.483 (0.210–1.111)
4–6	0.204	0.27	1.226 (0.569–2.643)
7 or more	0.302	0.55	1.352 (0.610–3.000)
Toxic leadership behaviors	−0.068^***^	117.69	0.935 (0.923–0.946)

B, multinomial-logit coefficient; Wald, Wald *χ*^2^ statistic; OR, odds ratio; CI, confidence interval. ^*^p < 0.05, ^**^p < 0.01, ^***^p < 0.001.

### 3.6 Statistical indirect associations through regulatory focus profiles

Covariates were selected based on their preliminary associations with career resilience or regulatory focus profile membership, as well as on evidence from previous literature. The final model included age, educational level, nursing experience, monthly night-shift frequency, and physical exercise. Uncertainty in the classification of profile membership was taken into account when assessing statistical indirect associations. Overall, a significant negative statistical indirect association was observed between toxic leadership behaviors and career resilience through the distribution of regulatory focus profiles (Table 6). Compared with the Low-Regulatory profile, no statistically significant relative indirect contribution was observed for the balanced profile, while the Prevention-Dominant and Dual-High profiles exhibited positive and negative relative indirect contributions, respectively. There was a significant difference in the profile-specific relative indirect contributions, Wald’s *χ*^2^(3) = 73.71, *p* < 0.001.

**Table 6:** Component paths and statistical indirect associations between toxic leadership behaviors and career resilience through regulatory focus profiles.

Association	Est.	SE	95% CI
Toxic leadership behaviors associated with profile membership			
Balanced	−1.573	0.171	[−1.907, −1.238]
Prevention-Dominant	−1.169	0.202	[−1.564, −0.773]
Dual-High	−1.887	0.174	[−2.227, −1.546]
Profile membership associated with career resilience			
Balanced	0.829	0.105	[0.648, 0.999]
Prevention-dominant	1.387	0.108	[1.167, 1.590]
Dual-high	1.582	0.107	[1.372, 1.770]
Overall associations			
Total association	−0.388	0.026	[−0.439, −0.344]
Direct association	−0.280	0.023	[−0.325, −0.241]
Overall indirect association	−0.108	0.017	[−0.145, −0.082]
Profile-specific relative indirect contributions			
Balanced	−0.010	0.014	[−0.029, 0.018]
Prevention-dominant	0.065	0.016	[0.040, 0.100]
Dual-high	−0.163	0.029	[−0.228, −0.116]

The low-regulatory profile was the reference category.

## 4 Discussion

This study examined the Association between toxic leadership behaviors and career resilience among nurses; additionally, statistical indirect associations involving regulatory focus profile membership were examined. Using LPA, four regulatory focus profiles among nurses were identified: Low-Regulatory, Balanced, Prevention-Dominant, and Dual-High. The Balanced profile was the most common (40.4%), followed by the Dual-High profile (37.9%). Toxic leadership behaviors were negatively associated with nurses’ career resilience. Furthermore, the different regulatory focus profiles exhibited different statistical indirect association patterns in this context. Specifically, compared with the Low-Regulatory profile, the Dual-High profile showed the strongest negative relative indirect contribution, while the Prevention-Dominant profile showed a weak but statistically significant positive relative indirect contribution. The findings suggest that the statistical indirect associations between toxic leadership behaviors and career resilience may differ according to nurses’ regulatory focus profile membership, reflecting different empirical configurations across promotion and prevention focus indicators.

This study found that toxic leadership behaviors of nurse managers are negatively associated with nurses’ career resilience. Previous studies have shown that destructive leadership behaviors—including authoritarian and abusive practices—can create unfavorable workplace environments characterized by reduced psychological safety and increased occupational strain (Labrague, 2024; Lavoie-Tremblay et al., 2016). Such environments are associated with emotional exhaustion, burnout, reduced job satisfaction, and other adverse occupational outcomes among nurses (Ofei et al., 2023; Labrague, 2024). Leadership behaviors represent important organizational resources or stressors that are associated with nurses’ perceptions of available support and psychological resources (Niinihuhta and Häggman-Laitila, 2022; Hobfoll, 2001). From a conservation-of-resources perspective, an unsupportive work environment can be viewed as a threat to valuable psychological resources, which may make adaptation to occupational adversity more difficult (Hobfoll, 2001). According to previous research, toxic leadership behaviors are associated with increased turnover intention (Ofei et al., 2023; Labrague, 2024), while a supportive work environment is associated with better resilience-related outcomes among nurses (Montgomery and Patrician, 2022).

In addition, this study also shows that nurses aged 30 years or older or with 10 or more years of nursing experience demonstrated greater career resilience than younger nurses or those with less experience. This finding is consistent with previous evidence indicating differences in nurses’ career resilience according to age and years of nursing experience (Qin et al., 2026). Accumulated clinical experience may be associated with greater clinical competence and more effective responses to complex practice situations (Benner, 1984). This may partly correspond to the higher career resilience observed among more experienced nurses. Interestingly, nurses with a master’s degree reported a lower level of career resilience than those with an associate or a bachelor’s degree. One possible explanation for this is that nurses with higher educational attainment may experience a greater discrepancy between their career expectations and the professional development opportunities available in their work environment. This mismatch may be associated with their perceptions of career resilience. However, this explanation is speculative and requires confirmation in longitudinal studies. Finally, nurses who exercise regularly report higher levels of career resilience than those who do not. Physical activity has been associated with a lower risk of incident depression (Schuch et al., 2018); exercise interventions among nurses have shown potential benefits for stress, burnout, anxiety, and psychological well-being (Wei et al., 2025). These psychological benefits may, in turn, support nurses’ adaptive functioning when they encounter career-related challenges.

We identified four regulatory focus profiles among nurses: the Low-Regulatory, Balanced, Prevention-Dominant, and Dual-High profiles. These profiles reflected a combination of overall level and configuration differences, rather than representing four entirely distinct motivational types. Among them, the Balanced (40.4%) and Dual-High (37.9%) profiles accounted for the largest proportions, indicating that these two response configurations were relatively common within the study sample. One possible interpretation is that nursing practice may involve both advancement-oriented demands, such as professional development and achievement, and prevention-oriented demands, such as safety, responsibility, and error avoidance. The coexistence of these demands may be compatible with the simultaneous activation of promotion- and prevention-focused tendencies (Higgins, 1997; Wallace et al., 2009; Shi et al., 2015; Wang et al., 2026). The Balanced and Prevention-Dominant profiles showed relatively similar mean levels of promotion focus, whereas the Prevention-Dominant profile showed a higher mean level of prevention focus. Consistent with Regulatory Focus Theory, this pattern suggests that nurses in the Prevention-Dominant profile may retain some advancement-oriented motivation while placing relatively greater emphasis on safety, responsibility, and the avoidance of undesirable outcomes (Higgins, 1997; Wallace et al., 2009). Older nurses had higher relative odds of Prevention-Dominant and Dual-High profile membership. One possible explanation is that accumulated professional experience may be accompanied by greater clinical competence and familiarity with the responsibilities of safe clinical practice (Benner, 1984), which may contribute to greater salience of both achievement- and prevention-oriented goals (Higgins, 1997; Wallace et al., 2009). Higher educational attainment was associated with lower relative odds of Prevention-Dominant and Dual-High profile membership compared with the Low-Regulatory profile. Nurses with lower educational attainment may have relatively salient advancement-related goals, such as pursuing further education or strengthening professional qualifications, although this explanation should be regarded as hypothesis-generating rather than established. Night-shift frequency was also associated with Prevention-Dominant profile membership. The higher relative odds observed in some night-shift categories may reflect the greater salience of vigilance and error-avoidance goals under night-shift conditions, as maintaining attention and psychomotor vigilance is particularly important for nurses working at night (Çolak and Esin, 2024).

In terms of career resilience, the Dual-High profile scored higher on this measure than the other three profiles. These two motivational orientations may act in a complementary way: the promotion-focused orientation may support professional development and efforts to pursue achievement, while the prevention-focused orientation may support a focus on safety, responsibility, and preventing errors (Higgins, 1997; Wallace et al., 2009). Therefore, their coexistence may provide a broader motivational foundation for coping with diverse career demands (Higgins, 1997; Wallace et al., 2009). However, the relatively higher scores for the Prevention-Dominant profile in terms of career resilience suggest that a prevention-dominant configuration can coexist with, rather than necessarily limit, career resilience. Given that prevention focus emphasizes safety, responsibility, vigilance, and avoiding negative consequences (Wallace et al., 2009), this orientation may be compatible with maintaining adaptive occupational functioning in nursing contexts; however, this interpretation remains tentative.

The results show that the statistical indirect association between toxic leadership behaviors and career resilience varies according to the regulatory focus profile. Compared with the Low-Regulatory profile, the negative relative indirect contribution was observed in the Dual-High profile. It is unlikely that this trend is solely due to prevention focus. Although the Prevention-Dominant profile showed relatively high prevention focus scores, its association pattern is different. The Dual-High profile reflects the simultaneous salience of advancement- and security-oriented goals. Individuals associated with this profile are highly focused on professional achievement while also being attentive to responsibilities, errors, and safety. However, maintaining goals for both advancement and security can be more challenging in a toxic leadership environment, which may partly explain the lower relative likelihood of Dual-High membership associated with higher levels of toxic leadership behaviors in this study. Leader behaviors serve as critical contextual cues that shape followers’ regulatory focus and their responses to workplace demands (Hamstra et al., 2014; Johnson et al., 2021). Toxic leadership has been associated with lower perceived support and unfavorable workplace outcomes (Labrague et al., 2020; Labrague, 2024; Hobfoll, 2001). Consequently, the lack of recognition, support, and autonomy not only hinders advancement-related aspirations but also increases concerns about fulfilling professional duties and avoiding negative outcomes (London, 1993; Hobfoll, 2001; Johnson et al., 2021). From a conservation-of-resources perspective, threats to valued psychological, social, and occupational resources may make sustained adaptation to demanding work conditions more difficult (Hobfoll, 2001). For nurses aiming to advance their careers while maintaining safety, such resource threats may be particularly relevant to the pursuit of both sets of goals (Higgins, 1997; Wallace et al., 2009). Repeated exposure to resource-threatening conditions may contribute to continued resource loss and limited opportunities for resource replenishment (Hobfoll, 2001).

Although the Prevention-Dominant profile showed a weak but statistically significant positive relative indirect contribution, it should not be taken as proof of a protective effect. One conceptual justification is that a prevention-focused orientation may be compatible with some of the core demands of nursing practice. Prevention focus places great importance on vigilance, responsibility, safety, and the avoidance of undesirable outcomes (Higgins, 1997; Wallace et al., 2009). These qualities are particularly relevant to clinical work, where patient safety and adherence to professional standards are paramount. Previous research has shown that a prevention focus in the workplace is associated with safety-related performance in situations where vigilance and error prevention are critical (Wallace et al., 2009). Therefore, nurses who demonstrate a Prevention-Dominant pattern may be better oriented toward maintaining established responsibilities and professional standards when faced with workplace uncertainty or adversity. However, because a prevention focus prioritizes safety and the fulfillment of obligations over development and growth, its potential adaptive value may depend on the occupational outcome under consideration. Therefore, a Prevention-Dominant pattern may be more closely aligned with maintaining occupational functioning under demanding conditions than with facilitating longer-term career advancement.

The negative association between toxic leadership behaviors and career resilience highlights the importance of supportive leadership and favorable work conditions, as well as the need to identify and address potentially harmful leadership behaviors. By strengthening leadership monitoring and development and improving feedback mechanisms and organizational support systems, hospital leaders can identify problematic leadership behaviors and attempt to address them (Niinihuhta and Häggman-Laitila, 2022; Labrague, 2024). The various regulatory focus profiles identified in this study suggest that interventions to support career resilience may benefit from greater flexibility rather than relying on predetermined methods. Rather than indicating fixed personal traits, these profiles may provide a basis for future research examining whether professional development, feedback, and support strategies can be adapted to nurses’ varying promotion- and prevention-focused motivational tendencies (Higgins, 1997; Johnson et al., 2021). Newly qualified nurses may particularly benefit from additional guidance and professional support, especially when transitioning to clinical practice (Gagnon-Béland et al., 2024). At the individual level, wellness initiatives, including opportunities for physical activity, may complement these organizational strategies, given evidence linking physical activity and exercise interventions with better psychological well-being and lower psychological distress (Schuch et al., 2018; Wei et al., 2025).

## 5 Limitations

Although this study provides a detailed analysis of the relationships between toxic leadership behaviors, regulatory focus profiles, and career resilience, there are some limitations that should be noted. First, the cross-sectional design of the study precludes drawing causal conclusions and does not allow for determining the temporal ordering of the observed relationships. Longitudinal studies are necessary to assess the stability and development of regulatory focus profiles and career resilience over time. Second, all variables were measured using self-reported data, which may be subject to social desirability bias, recall bias, and common-method variance. Although validated instruments were used, measurement error cannot be completely excluded. Future research should use more diverse samples and, where possible, include data from multiple sources or objective measures. In addition, the use of convenience sampling and recruitment from six tertiary hospitals in three Chinese provinces may limit the generalizability of the findings to nurses in other regions or healthcare settings. Third, potential clustering of nurses within hospitals could not be accounted for because hospital-level identifiers were unavailable in the analytic dataset, which may have affected the precision of the estimated standard errors. Finally, latent profile solutions are sample-dependent, and profile membership is probabilistic rather than certain. Although classification uncertainty was addressed using error-corrected procedures, the use of assigned profiles in subsequent analyses may still lead to residual classification error. Therefore, the identified profiles should be considered not as fixed psychological categories, but as sample-specific statistical response patterns. The findings also depend on the assumptions and model specifications underlying the LPA and subsequent analyses; alternative specifications may yield somewhat different profile solutions or association estimates. In order to assess the stability and generalizability of the profile structure and profile-specific associations, it is necessary to replicate the findings in independent samples.

## 6 Conclusion

This study was conducted on a sample of 1,210 nurses. Using LPA and profile-based indirect association analysis, the associations among toxic leadership behaviors, regulatory focus profiles, and career resilience were examined. The results revealed a moderate negative association between toxic leadership behaviors and career resilience. Furthermore, it was observed that the different regulatory focus profiles showed different statistical indirect association patterns. Four regulatory focus profiles were identified among the nurses: Low-Regulatory, Balanced, Prevention-Dominant, and Dual-High. Relative to the Low-Regulatory profile, a negative profile-specific relative indirect contribution was observed for the Dual-High profile. Specifically, higher toxic leadership behaviors were associated with lower odds of membership in the Dual-High profile, and the BCH-estimated career resilience contrast for this profile contributed to the observed negative relative indirect association. Hospital managers should therefore be aware of toxic leadership behaviors and promote psychologically safe and supportive work environments, which may support nurses’ career resilience. Although regulatory focus profiles may provide a potential framework for understanding individual differences among nurses, whether profile-based support strategies improve outcomes requires further investigation.

## Data Availability

The raw data supporting the conclusions of this article will be made available by the authors, without undue reservation.
