# Supplementary material for: The association between toxic leadership behaviors and nurses’ career resilience: heterogeneous indirect roles of regulatory focus profiles
Source: Front Psychol. 2026 Sep 7;17:1951423. doi: 10.3389/fpsyg.2026.1951423 (PMC13590365; doi:10.3389/fpsyg.2026.1951423)
Supplement: Supplementary file 1 [file Table_1.DOCX]

**Table S1** Univariable multinomial logistic regression analyses of factors associated with regulatory focus profile membership

| Variables | Balanced vs. Low-Regulatory | | | Prevention-Dominant vs. Low-Regulatory | | |
| --- | --- | --- | --- | --- | --- | --- |
|  | B | Wald | OR (95% CI) | B | Wald | OR (95% CI) |
| Age (years) |  |  |  |  |  |  |
| < 30 | Ref. |  |  | Ref. |  |  |
| ≥30 | 0.909^**^ | 10.82 | 2.48 (1.44–4.27) | 1.394^***^ | 20.45 | 4.03 (2.20–7.38) |
| Gender |  |  |  |  |  |  |
| Male | Ref. |  |  | Ref. |  |  |
| Female | 0.327 | 1.23 | 1.39 (0.78–2.47) | −0.405 | 1.54 | 0.67 (0.35–1.27) |
| Education level |  |  |  |  |  |  |
| Associate degree | Ref. |  |  | Ref. |  |  |
| Bachelor's degree | 1.133^**^ | 8 | 3.11 (1.42–6.81) | 0.278 | 0.40 | 1.32 (0.56–3.13) |
| Master's degree or above | 0.512 | 1.2 | 1.67 (0.67–4.17) | 0.015 | <0.01 | 1.02 (0.36–2.84) |
| Parental status |  |  |  |  |  |  |
| No | Ref. |  |  | Ref. |  |  |
| Yes | 0.233 | 1.01 | 1.26 (0.80–1.99) | 0.246 | 0.76 | 1.28 (0.73–2.23) |
| Nursing experience (years) |  |  |  |  |  |  |
| ≤5 | Ref. |  |  | Ref. |  |  |
| 6-9 | 0.373 | 1.71 | 1.45 (0.83–2.54) | 0.838^*^ | 6.44 | 2.31 (1.21–4.41) |
| ≥10 | 1.197^***^ | 11.91 | 3.31 (1.68–6.53) | 1.474^***^ | 14.69 | 4.37 (2.06–9.28) |
| Monthly night shifts |  |  |  |  |  |  |
| 0 | Ref. |  |  | Ref. |  |  |
| 1-3 | −0.252 | 0.54 | 0.78 (0.40–1.53) | −1.253^*^ | 5.48 | 0.29 (0.10–0.82) |
| 4-6 | −0.024 | 0.01 | 0.98 (0.52–1.82) | −0.492 | 1.36 | 0.61 (0.27–1.40) |
| 7 or more | 0.332 | 1.03 | 1.39 (0.73–2.64) | 1.084^**^ | 7.83 | 2.96 (1.38–6.32) |
| Clinical Department |  |  |  |  |  |  |
| Critical Care Units | Ref. |  |  | Ref. |  |  |
| Surgical | −0.185 | 0.33 | 0.83 (0.44–1.57) | −0.358 | 0.91 | 0.70 (0.34–1.46) |
| Medical | −0.536 | 2.77 | 0.58 (0.31–1.10) | −0.567 | 2.28 | 0.57 (0.27–1.18) |
| Other departments | −0.304 | 0.66 | 0.74 (0.35–1.54) | −0.538 | 1.45 | 0.58 (0.24–1.40) |
| Physical exercise |  |  |  |  |  |  |
| Never exercises | Ref. |  |  | Ref. |  |  |
| Exercises occasionally | 0.026 | 0.01 | 1.03 (0.66–1.59) | −0.020 | 0.01 | 0.98 (0.58–1.66) |
| Exercises regularly | 0.844 | 3.46 | 2.33 (0.96–5.66) | 0.7 | 1.85 | 2.01 (0.74–5.51) |
| Toxic leadership behaviors | −0.052^***^ | 98.89 | 0.949 (0.939–0.959) | −0.041^***^ | 52.28 | 0.960 (0.949–0.970) |
| Variables | Dual-High vs. Low-Regulatory | | |  |  |  |
|  | B | Wald | OR (95% CI) |  |  |  |
| Age (years) |  |  |  |  |  |  |
| < 30 | Ref. |  |  |  |  |  |
| ≥30 | 1.762^***^ | 40.38 | 5.82 (3.38–10.03) |  |  |  |
| Gender |  |  |  |  |  |  |
| Male | Ref. |  |  |  |  |  |
| Female | 0.343 | 1.28 | 1.41 (0.78–2.55) |  |  |  |
| Education level |  |  |  |  |  |  |
| Associate degree | Ref. |  |  |  |  |  |
| Bachelor's degree | 0.179 | 0.26 | 1.20 (0.60–2.40) |  |  |  |
| Master's degree or above | −1.582^**^ | 9.49 | 0.21 (0.08–0.56) |  |  |  |
| Parental status |  |  |  |  |  |  |
| No | Ref. |  |  |  |  |  |
| Yes | 0.095 | 0.16 | 1.10 (0.69–1.74) |  |  |  |
| Nursing experience (years) |  |  |  |  |  |  |
| ≤5 | Ref. |  |  |  |  |  |
| 6-9 | 0.624^*^ | 4.39 | 1.87 (1.04–3.35) |  |  |  |
| ≥10 | 2.086^***^ | 36.4 | 8.05 (4.09–15.85) |  |  |  |
| Monthly night shifts |  |  |  |  |  |  |
| 0 | Ref. |  |  |  |  |  |
| 1-3 | −0.821^*^ | 5.43 | 0.44 (0.22–0.88) |  |  |  |
| 4-6 | −0.574 | 3.21 | 0.56 (0.30–1.06) |  |  |  |
| 7 or more | 0.094 | 0.08 | 1.10 (0.58–2.07) |  |  |  |
| Clinical Department |  |  |  |  |  |  |
| Critical Care Units | Ref. |  |  |  |  |  |
| Surgical | −0.050 | 0.02 | 0.95 (0.50–1.82) |  |  |  |
| Medical | −0.413 | 1.56 | 0.66 (0.35–1.26) |  |  |  |
| Other departments | −0.247 | 0.41 | 0.78 (0.37–1.66) |  |  |  |
| Physical exercise |  |  |  |  |  |  |
| Never exercises | Ref. |  |  |  |  |  |
| Exercises occasionally | −0.042 | 0.03 | 0.96 (0.62–1.49) |  |  |  |
| Exercises regularly | 0.675 | 2.14 | 1.96 (0.80–4.85) |  |  |  |
| Toxic leadership behaviors | −0.063^***^ | 129.56 | 0.939 (0.929–0.949) |  |  |  |

**Note.** B = multinomial-logit coefficient; Wald = Wald χ² statistic; OR = odds ratio; CI = confidence interval. ^*^*p* < 0.05,  ^* *^*p* < 0.01,  ^* * *^*p* < 0.001.
